# Nutritional status, cognitive achievement, and educational attainment of children aged 8-11 in rural South India

**DOI:** 10.1371/journal.pone.0223001

**Published:** 2019-10-09

**Authors:** Yubraj Acharya, Nancy Luke, Marco Faytong Haro, Winsley Rose, Paul Swamidhas Sudhakar Russell, Anu Mary Oommen, Shantidani Minz

**Affiliations:** 1 Department of Health Policy and Administration, The Pennsylvania State University, Pennsylvania, United States of America; 2 Department of Sociology and Criminology, The Pennsylvania State University, Pennsylvania, United States of America; 3 Department of Pediatrics, Christian Medical College, Vellore, India; 4 Department of Psychiatry, Christian Medical College, Vellore, India; 5 Department of Community Health, Christian Medical College, Vellore, India; Indian Statistical Institute, INDIA

## Abstract

**Background:**

Malnutrition among children is one of the most pressing health concerns middle- and low-income countries face today, particularly those in Sub-Saharan Africa and South Asia. Early-life malnutrition has been shown to affect long-term health and income. One hypothesized channel linking early-life malnutrition and long-term outcomes is cognitive development. However, there is limited empirical evidence on the relationship between nutritional status and cognitive achievement in middle childhood.

**Study design:**

As part of the South India Community Health Study (SICHS), we collected educational attainment and anthropometric data from 1,194 children in rural Vellore district of Tamil Nadu, India, and assessed their math and reading skills. We analyzed the relationship between continuous and binary anthropometric measures of nutritional status and three measures of cognitive achievement (reading, math, and grade level), adjusting for potential confounders, using a regression framework.

**Results:**

Lower height-for-age and weight-for-age and their corresponding binary measures (stunting, underweight) were associated with lower reading scores, lower math scores, and lower grade level, with the exception of the association between weight-for-age and reading, which was marginally significant. A stunted child had one-third of a grade disadvantage compared to a non-stunted counterpart, whereas an underweight child had one-fourth of a grade disadvantage compared to a non-underweight counterpart. Lower BMI-for-age was associated with grade level and marginally associated with lower math scores, and its binary measure (thinness) was marginally associated with lower math scores.

**Conclusions:**

Acute and chronic malnutrition in middle childhood were negatively associated with math scores, reading scores, and educational attainment. Our study provides new evidence that cognitive achievement during middle childhood could be an important mechanism underlying the association between early-life malnutrition and long-term wellbeing.

## Introduction

Malnutrition among children is one of the most pressing health concerns low- and middle-income countries face today, particularly in Sub-Saharan Africa and South Asia. In 2016, an estimated 155 million children under age five were stunted and 52 million wasted worldwide [1]. A large body of research has shown that poor nutritional status in childhood has lasting effects into adulthood. For example, early-life nutrition is an important determinant of one’s long-term productivity, earnings, and health [2–5]. This evidence, coupled with the announcement of the Sustainable Development Goals, has prompted renewed efforts around the world to design and implement policies to address child malnutrition.

One hypothesized channel through which nutritional status affects long-term wellbeing is cognitive development. Existing research has examined this relationship using various indicators of nutritional status and cognitive achievement [6–14]. A number of these studies focused on the nutritional status of children below age five. This choice is not surprising given the critical role that early-life nutrition, particularly during the first 1,000 days of life, has been shown to have on cognitive development and subsequent educational attainment [15,16]. There is some evidence that the impact of early-life malnutrition on health could be partially reversible [3,8,17], supporting the view that efforts to address child nutrition could reverse, or at least mitigate, some of the effects of early-life nutritional deficiencies on cognitive development [9].

Despite this body of research, an important gap remains in our understanding of the nutrition-cognition nexus in later, potentially influential, years in childhood. Several recent studies have examined the nutrition-cognition nexus among children older than five years. For example, Peringon and colleagues studied the association between various nutritional deficiencies and cognitive achievement among Cambodian children aged 6–16 [18]. They found that stunted children performed significantly poorer compared to non-stunted children on several standardized tests. Using data from Indonesia, Malaysia, Thailand, and Vietnam, Sandjaja and colleagues also found that undernourishment and cognition (as measured by non-verbal intelligence quotients) were significantly associated among children ages 6–12 in these countries [19]. Likewise, Case and Paxson documented the association between height-for-age z-scores and cognitive ability in children aged 5 and 10 years in the United Kingdom and 7 and 11 years in the United States [20]. They found that, even among children born to the same mother, taller siblings (as measured by their height-for-age z-scores) scored better on a range of cognitive tests and progressed through school more quickly.

The current study examines the nutrition-cognition nexus among children aged 8–11 in rural Tamil Nadu, South India. The cross-sectional survey data include anthropometric information and measures of reading ability, mathematical ability, and educational attainment collected from 1,194 children. We constructed multiple indicators of nutritional status: z-scores for height-for-age, BMI-for-age, and weight-for-age, and corresponding binary measures of stunting, thinness (also called wasting for children aged 0–59 months), and underweight.

Two previous studies from India have examined the relationship between height-for-age, specifically, and cognitive achievement among school-age children using measures of reading and mathematical ability assessed from the Pratham’s Annual Status of Education Report (ASER) Survey [21]. The current study used these same measures of children’s reading and math, described below. Kingdon and Monk collected data from children in rural areas in 11 districts of India and found a positive association of height-for-age z-scores and reading and math scores among children aged 6–14 [22]. Likewise, using nationally representative data from India, Spears [23] found a positive association between height-for-age z-scores and ASER reading and math scores among children aged 8–11.

Stunting (height-for-age <-2 z scores), the outcome measure used in these previous studies in India, results from chronic malnutrition (inadequate nutrition over a long period) and therefore reflects the cumulative effect of past nutritional deficiencies [5,24]. We extended this research by investigating the impact of additional measures of nutritional deficiency, namely thinness and underweight, on cognitive achievement. Thinness (BMI-for-age <-2 z scores) originates from acute inadequate nutrition, which leads to rapid weight loss or failure to gain weight normally [24]. Underweight (weight-for-age <-2 z scores) is a combination measure that can occur because of stunting, thinness, or both [24]. From a policy point of view, research on acute and chronic child malnutrition can help target scarce resources toward appropriate responses to short- or longer-term issues, or both.

## Materials and methods

### Setting

Data from the South India Community Health Study (SICHS) were used for the analyses. SICHS was conducted in rural areas of Vellore district in Tamil Nadu, India. India is an appropriate site for this study given the high prevalence of malnutrition and poor educational outcomes. Indian children are among the most severely malnourished worldwide despite India’s rapid economic development. In 2016, 38% of Indian children under age five were stunted, 21% were thin, and 36% were underweight [25]. Corresponding figures for rural Tamil Nadu are 27% stunted, 20% thin, and 24% underweight [25]. In terms of educational indicators, although overall enrollment among children aged 6–14 is over 96% in India, learning outcomes remain poor in rural areas. For example, in 2018, only a quarter of all children in grade three were able to read text and perform math calculations expected at that grade level [26]. In addition, only 27% of children in grade three could read a grade two textbook, and only 28% could do grade two-level subtraction [26].

This study was approved by the Institutional Review Boards of the Pennsylvania State University, USA, and the Christian Medical College, Tamil Nadu, India.

### Study design

Between 2012 and 2014, the SICHS research team conducted a census of over 300,000 households in rural Vellore district, Tamil Nadu. A household survey was undertaken in 2015–16 in a sample of 5,000 households. The sampling frame for the household survey included all ever-married men aged 25–60 in the SICHS census (referred to as male primary respondents) plus a small number of divorced or widowed women (female primary respondents) with “missing" husbands who would have been aged 25–60, based on the average age-gap between husbands and wives in the census. The sample of primary respondents was subsequently drawn to be representative of each caste in the study area, excluding castes with less than 100 households in the census. The response rate for primary respondent households was 85%. The study area is representative of rural Tamil Nadu and rural South India with respect to socioeconomic and demographic characteristics [27].

Primary respondents or their spouses completed a household roster that included all of their resident children, and 1,313 children aged 8–11 were listed. To create the analytical sample for this study, we dropped 83 observations for children who did not complete the ASER reading and math survey, which provided information for our dependent variables. We dropped an additional 10 observations with missing information on weight and height, which was required to create the key independent variables. We also dropped 11 observations with extreme values on any of the anthropometric measures (less than or greater than five SDs). Finally, we removed 15 observations with missing information on any of the covariates. This yielded an analytic sample of 1,194 children aged 8–11.

### Outcome variables

We used the child’s scores in reading and mathematics and the current grade level as three dependent variables in the study. The tests for reading and mathematics were developed by Pratham, an Indian NGO, and are annually implemented in Pratham’s Annual Status of Education Report (ASER) Survey throughout India. The SICHS survey followed the same methodology as the ASER survey.

To measure reading level, a trained interviewer asked a child to read a paragraph at the grade one level (with four sentences and approximately 19 words) [21]. If the child could read the paragraph (an entire sentence rather than a string of words), he/she was asked to read a short story at grade two level (with seven to 10 sentences and 60 words). If the child could not read the paragraph, then he/she was asked to read any four out of the five words that were listed. If the child could not read at least four words, he/she was asked to identify any four out of the five letters that were listed. For the purpose of the analysis, we categorized the students into five groups: (1) those who could not identify at least four out of the five letters, (2) those who could identify at least four out of the five letters but not read at least four words, (3) those who could read at least four words but not the paragraph without making more than three mistakes, (4) those who could read the paragraph but not the short story without making more than three mistakes, and (5) those who could read the short story without making more than three mistakes. Following Spears [23], we coded these categories 1–5.

Similarly, for mathematics, the five categories were: those who could not recognize four out of any five single-digit numbers (coded as 1), those who could recognize at least four out of five single-digit numbers but not numbers 11 to 99 (coded as 2), those who could recognize at least four out of five double-digit numbers but could not solve a simple subtraction problem (two digit numerical problem with borrowing) (coded as 3), those who could solve the subtraction problem but not a division problem (three digit number divided by one digit number with a remainder) (coded 4), and those who could solve the division problem (coded 5). Finally, we asked mothers to report the current grade level of the child.

### Explanatory variables

The six key independent variables were anthropometric measures of nutritional status. Anthropometric information (height and weight) was collected for each child from a trained nurse or interviewer, and child age was reported by a parent. We used this information and the user-written zanthro program in Stata [28] to construct z-scores for height-for-age, BMI-for-age, and weight-for-age [29]. The zanthro program calculates each nutritional status measure based on the 2006 WHO Growth Charts [29], with the exception of weight-for-age for children age 11, which is calculated using UK WHO Growth Charts [30]. We also categorized children with height-for-age scores below two standard deviations from the reference median as stunted. We created similar binary variables for thinness (BMI-for-age z-score <-2 SD) and underweight (weight-for-age z-score <-2 SD).

In order to reduce bias from potential confounders, we controlled for several factors that have been found to influence cognition and nutritional status. Information for each of these variables was collected from children’s mothers or fathers. Discrimination against girls in South Asian societies and its implications for girls’ health has been widely documented [31–35]. In these societies, gender plays an important role in determining access to food, health services, and educational resources, with a pronounced bias against girls. It is also reasonable to expect cognition and nutritional status to change with a child’s age. Therefore, we controlled for age and gender of the child.

Recognizing that cognitive development could be influenced by a child’s physical and mental health conditions, including birth defects or other disabilities, we generated a binary measure for each child indicating if a parent reported that the child had any chronic illness or congenital or perinatal disorder. This variable was set to one for children who had at least one of the following conditions: any bodily deformity, heart disease/defects, diabetes, hypertension, asthma, thyroid, epilepsy, mental illness, or any other chronic condition.

We also controlled for mother’s education, as previous studies have shown this to be a strong predictor of children’s health outcomes [36–38]. Likewise, we controlled for father’s education level. In order to account for differences in caregiving by family structure, we controlled for whether the child lived with both biological parents or with a single parent or others. We controlled for household’s monthly income and the language in which the child took the ASER test as proxies for the household’s economic status (English vs. Tamil). Household income was calculated as the sum of two sources: information on wages and salaries of all household members in the last month; and household income earned from land and livestock in the last year, divided by 12 to produce a monthly average. In India, children who attend English medium schools tend to be from higher economic status than those who attend Hindi or local language medium schools. To capture food insecurity, we controlled for whether the mother reported being worried about running out of food due to lack of money during the month preceding the survey. Finally, we controlled for father’s caste or tribe to account for underlying differences in access to resources as well as cultural behaviors, such as feeding practices, between individuals from different groups [39,40]. We coded caste/tribe dichotomously as scheduled caste/ scheduled tribe (historically disadvantaged groups) or not.

### Statistical analysis

In order to assess the relationship between a child’s nutritional status and cognitive achievement, we estimated regressions of the following form:

$$Y_{ij}=\alpha_{m}+\beta Z_{ij}+\theta X_{ij}+\varepsilon_{ij}$$ Eq (1)

In Eq (1), Y_*ij*_ is the reading score, mathematics score, or the grade level of the child *i* in household *j*. Z_*ij*_ is the measure of nutritional status. We estimated regression models using continuous (i.e., z-scores for height-for-age, weight-for-age, and BMI-for-age) as well as binary indicators (i.e., stunted, underweight, and thin). **X** refers to the vector of potential confounders mentioned above. ε is the usual error term.

Given that reading and mathematics scores are ordinal variables, we followed Spears (2012) [23] and estimated the relationship between nutritional status and these outcomes using ordered logit regression. When the child’s grade level is the outcome, we used ordinary least squares (OLS), in which case α_*m*_ is the mean of the outcome for the reference group. In all cases, we clustered the standard errors at the level of the household, thus allowing arbitrary correlation in the outcome for multiple children from the same household.

The coefficient or the odds ratio β reflects the association between nutritional status and the outcomes. The expected sign of the coefficient or the value of the odds ratio depends on the measure of nutritional status used in the regression. When the measure is stunting (binary), for example, we expected the odds ratio β < 1 because stunted children are expected to have poorer cognitive achievement than children who are not stunted. When the measure is height-for-age z-scores (a continuous measure), on the other hand, we expected the coefficient β > 0.

The statistical significance of associations is reported at the *P*<0.1, *P*<0.05, *P*<0.01, and *P*<0.001 levels. All analyses were carried out using the Stata statistical software package version 15 [41].

## Results

### Descriptive statistics

The average child aged 8–11 in our sample had completed grade four and had a reading score of 3.9, meaning the child was close to being able to read a paragraph (**Table 1**). The average child was close to being able to perform simple division, as the average math score (3.8) suggests. The average height-for-age was 0.6 standard deviation below the median of the reference population. The average BMI-for-age and weight-for-age were approximately one standard deviation below the reference median. As such, 9.6% of children in the sample were stunted, 23.9% were thin, and 24.9% children were underweight.

**Table 1:** Children aged 8–11 outcome measures, anthropometrics, and socio-demographic characteristics (N = 1,194).

Variable	Obs	Mean or %	S.d.	Range
**Outcome variables**				
Math score		3.77	0.90	1–5
Reading score		3.87	1.28	1–5
Grade level		4.46	1.35	0–8
**Key explanatory variables**				
Height-for-age z-score		-0.58	1.09	-4.55–3.58
BMI-for-age z-score		-1.01	1.42	-4.93–3.79
Weight-for-age z-score		-1.09	1.31	-4.91–3.45
Stunted (binary)		9.63%		
Thinness (binary)		23.95%		
Underweight (binary)		24.96%		
**Control variables**				
Child age				
8 years old	266	22.28%		
9 years old	276	23.12%		
10 years old	304	25.46%		
11 years old	348	29.15%		
Girl		48.58%		
Both biological parents in household		87.02%		
Father's education				
No education	153	12.81%		
Primary	496	41.54%		
Secondary	480	40.20%		
Post-secondary	65	5.44%		
Mother's education				
No education	188	15.75%		
Primary	508	42.55%		
Secondary	457	38.27%		
Post-secondary	41	3.43%		
Household income (thousands of Rupees)		8.50	7.95	0–89
Scheduled caste/tribe		28.98%		
English medium school		27.55%		
Worried about not having sufficient food during the past month		24.29%		
Any chronic disease, or congenital or perinatal disorder		2.93%		

In the sample, 48.6% of children were girls, and 87% lived with both biological parents. The majority of children’s fathers and mothers had primary-level education or no education at all. The average monthly income was Indian Rupees (IRs) 8,500 (range 0–89,000) (the exchange rate at the time of the survey was approximately 1 US dollar to 65 IRs). Nearly 29% of the children were from scheduled castes or tribes and 27.6% of children took the reading and mathematics tests in English. Nearly 25% of children lived in households that reported having worried about not having enough food to eat in the month preceding the survey, and 2.9% had at least one chronic disease or congenital or perinatal disorder.

### Results from multivariate analysis

Odds ratios for the ordered logit analysis, coefficients for the OLS analysis, and standard errors from the regression estimating Eq (1) are presented in Tables 2 (for height-for-age and stunting), 3 (for BMI-for-age and thinness), and 4 (for weight-for-age and underweight). For comparison, we report the odds ratios (or coefficients) and standard errors from bivariate regressions—i.e., without controlling for the potential confounders mentioned above—in **S1 Table**.

**Table 2:** Adjusted models for the association between cognitive achievement and educational attainment and height-for-age z-scores and stunting.

Outcome →	Math score		Reading score		Grade level	
	Odds ratios		Odds ratios		OLS regression coefficients	
Height-for-age	1.117*		1.123*		0.142***	
	(0.058)		(0.063)		(0.022)	
Stunted		0.612*		0.663*		-0.323***
		(0.128)		(0.132)		(0.089)
Child age (*Ref = 8 years*)						
9 years old	2.078***	2.067***	1.697***	1.683***	1.040***	1.030***
	(0.316)	(0.314)	(0.267)	(0.264)	(0.057)	(0.058)
10 years old	3.222***	3.257***	2.698***	2.708***	2.049***	2.047***
	(0.500)	(0.505)	(0.405)	(0.406)	(0.057)	(0.058)
11 years old	3.619***	3.650***	3.365***	3.372***	3.010***	3.003***
	(0.557)	(0.563)	(0.508)	(0.511)	(0.058)	(0.058)
Girl	1.467***	1.467***	2.053***	2.055***	0.099*	0.094*
	(0.166)	(0.166)	(0.235)	(0.236)	(0.041)	(0.042)
Both biological parents in hh	1.203	1.205	1.245	1.233	-0.056	-0.056
(*Ref = Only one bio*. *parent in hh*)	(0.222)	(0.222)	(0.204)	(0.201)	(0.061)	(0.061)
Father’s education (*Ref = Secondary education*)						
No education	0.892	0.861	0.810	0.782	-0.080	-0.117
	(0.202)	(0.195)	(0.161)	(0.155)	(0.089)	(0.089)
Primary education	1.218	1.210	0.981	0.978	-0.058	-0.057
	(0.159)	(0.158)	(0.133)	(0.132)	(0.053)	(0.054)
Post-secondary education	1.414	1.424	1.105	1.115	0.008	0.021
	(0.349)	(0.350)	(0.298)	(0.298)	(0.096)	(0.099)
Mother’s education (*Ref = Secondary education*)						
No education	0.686+	0.695+	0.867	0.872	-0.117	-0.109
	(0.139)	(0.142)	(0.166)	(0.167)	(0.074)	(0.076)
Primary education	1.256+	1.286+	1.440**	1.472**	-0.088+	-0.064
	(0.166)	(0.168)	(0.199)	(0.201)	(0.051)	(0.052)
Post-secondary education	2.319**	2.356**	2.647**	2.705**	-0.267+	-0.239+
	(0.673)	(0.676)	(0.845)	(0.854)	(0.140)	(0.142)
HH income (thousands of Rupees)	1.009	1.010	1.017*	1.018*	-0.002	-0.000
	(0.007)	(0.007)	(0.008)	(0.008)	(0.002)	(0.002)
Scheduled caste/tribe	0.647**	0.648**	0.784+	0.786+	0.014	0.011
	(0.091)	(0.090)	(0.102)	(0.102)	(0.052)	(0.053)
English medium school	1.045	1.049	0.342***	0.345***	-0.216***	-0.204***
	(0.135)	(0.136)	(0.048)	(0.048)	(0.053)	(0.053)
Worried about not having	0.907	0.898	0.720*	0.714*	-0.063	-0.069
sufficient food during the past month	(0.133)	(0.132)	(0.102)	(0.101)	(0.059)	(0.059)
Any chronic disease, or congenital	1.138	1.171	1.166	1.202	0.219+	0.244*
or perinatal disorder	(0.317)	(0.317)	(0.432)	(0.442)	(0.114)	(0.115)
Constant					3.078***	3.012***
					(0.088)	(0.089)
Observations	1,194	1,194	1,194	1,194	1,194	1,194
R squared					0.710	0.702

Robust standard errors in parentheses.

*** p<0.001

** p<0.01

* p<0.05

+ p<0.1

**Table 3:** Adjusted models for the association between cognitive achievement and educational attainment and BMI-for-age z-scores and thinness.

Outcome →	Math score		Reading score		Grade level	
	Odds ratios		Odds ratios		OLS regression coefficients	
BMI-for-age	1.075+		1.027		0.040*	
	(0.043)		(0.043)		(0.016)	
Thinness		0.769+		0.862		-0.069
		(0.107)		(0.117)		(0.055)
Child age (*Ref = 8 years*)						
9 years old	2.032***	2.068***	1.662**	1.677***	1.019***	1.025***
	(0.309)	(0.315)	(0.260)	(0.262)	(0.058)	(0.058)
10 years old	3.188***	3.209***	2.656***	2.672***	2.033***	2.034***
	(0.493)	(0.497)	(0.396)	(0.399)	(0.058)	(0.059)
11 years old	3.506***	3.593***	3.277***	3.327***	2.980***	2.988***
	(0.539)	(0.553)	(0.495)	(0.504)	(0.058)	(0.059)
Girl	1.420**	1.425**	2.020***	2.014***	0.075+	0.083+
	(0.160)	(0.161)	(0.232)	(0.232)	(0.043)	(0.043)
Both biological parents in hh	1.220	1.221	1.240	1.242	-0.050	-0.052
(*Ref = Only one bio parent in hh*)	(0.226)	(0.226)	(0.204)	(0.205)	(0.062)	(0.062)
Father’s education (*Ref = Secondary education*)						
No education	0.876	0.873	0.787	0.789	-0.111	-0.115
	(0.199)	(0.199)	(0.157)	(0.157)	(0.090)	(0.090)
Primary education	1.222	1.228	0.988	0.990	-0.051	-0.046
	(0.158)	(0.159)	(0.135)	(0.134)	(0.054)	(0.054)
Post-secondary education	1.393	1.398	1.119	1.114	0.014	0.024
	(0.342)	(0.341)	(0.302)	(0.298)	(0.098)	(0.098)
Mother’s education (*Ref = Secondary education*)						
No education	0.690+	0.681+	0.865	0.859	-0.112	-0.116
	(0.141)	(0.138)	(0.168)	(0.167)	(0.078)	(0.078)
Primary education	1.269+	1.264+	1.474**	1.469**	-0.069	-0.065
	(0.166)	(0.165)	(0.201)	(0.200)	(0.052)	(0.052)
Post-secondary education	2.394**	2.389**	2.753**	2.752**	-0.227	-0.228
	(0.675)	(0.676)	(0.877)	(0.881)	(0.143)	(0.142)
HH income (thousands of Rupees)	1.010	1.010	1.018*	1.018*	-0.000	0.000
	(0.007)	(0.007)	(0.008)	(0.008)	(0.002)	(0.002)
Scheduled caste/tribe	0.640**	0.648**	0.785+	0.789+	0.006	0.012
	(0.090)	(0.090)	(0.103)	(0.103)	(0.053)	(0.053)
English medium school	1.033	1.052	0.346***	0.348***	-0.209***	-0.198***
	(0.133)	(0.136)	(0.048)	(0.048)	(0.054)	(0.054)
Worried about not having	0.909	0.898	0.726*	0.725*	-0.056	-0.060
sufficient food during the past month	(0.133)	(0.132)	(0.103)	(0.103)	(0.060)	(0.060)
Any chronic disease, or congenital	1.136	1.156	1.156	1.166	0.212+	0.218+
or perinatal disorder	(0.310)	(0.317)	(0.417)	(0.423)	(0.112)	(0.113)
Constant					3.036***	2.998***
					(0.089)	(0.088)
Observations	1,194	1,194	1,194	1,194	1,194	1,194
R squared					0.699	0.698

Robust standard errors in parentheses.

*** p<0.001

** p<0.01

* p<0.05

+ p<0.1

**Table 4:** Adjusted models for the association between cognitive achievement and educational attainment and weight-for-age z-scores and underweight.

Outcome →	Math score		Reading score		Grade level	
	Odds ratios		Odds ratios		OLS regression coefficients	
Weight-for-age	1.130**		1.086+		0.100***	
	(0.051)		(0.052)		(0.018)	
Underweight		0.665**		0.714*		-0.241***
		(0.088)		(0.095)		(0.052)
Child age (*Ref = 8 years*)						
9 years old	2.039***	2.035***	1.660**	1.655**	1.018***	1.018***
	(0.310)	(0.309)	(0.260)	(0.259)	(0.057)	(0.057)
10 years old	3.183***	3.187***	2.647***	2.659***	2.030***	2.032***
	(0.494)	(0.493)	(0.396)	(0.397)	(0.057)	(0.058)
11 years old	3.460***	3.517***	3.234***	3.272***	2.964***	2.976***
	(0.532)	(0.538)	(0.491)	(0.495)	(0.058)	(0.058)
Girl	1.427**	1.415**	2.010***	1.996***	0.069+	0.072+
	(0.161)	(0.160)	(0.230)	(0.229)	(0.042)	(0.042)
Both biological parents in hh	1.217	1.213	1.244	1.236	-0.051	-0.055
(*Ref = Only one bio parent in hh*)	(0.225)	(0.224)	(0.205)	(0.202)	(0.061)	(0.061)
Father’s education (*Ref = Secondary education*)						
No education	0.893	0.886	0.800	0.800	-0.091	-0.102
	(0.202)	(0.199)	(0.158)	(0.158)	(0.089)	(0.088)
Primary education	1.208	1.221	0.976	0.987	-0.061	-0.047
	(0.157)	(0.159)	(0.134)	(0.134)	(0.053)	(0.053)
Post-secondary education	1.374	1.419	1.095	1.118	-0.006	0.024
	(0.338)	(0.345)	(0.295)	(0.297)	(0.099)	(0.098)
Mother’s education (*Ref = Secondary education*)						
No education	0.690+	0.692+	0.866	0.874	-0.113	-0.110
	(0.140)	(0.141)	(0.167)	(0.170)	(0.076)	(0.077)
Primary education	1.250+	1.260+	1.448**	1.451**	-0.086+	-0.076
	(0.164)	(0.165)	(0.199)	(0.199)	(0.051)	(0.051)
Post-secondary education	2.343**	2.273**	2.701**	2.623**	-0.246+	-0.259+
	(0.665)	(0.653)	(0.868)	(0.841)	(0.142)	(0.143)
HH income (thousands of Rupees)	1.010	1.010	1.017*	1.017*	-0.001	-0.001
	(0.007)	(0.007)	(0.008)	(0.008)	(0.002)	(0.002)
Scheduled caste/tribe	0.638**	0.647**	0.781+	0.789+	0.004	0.012
	(0.090)	(0.090)	(0.102)	(0.103)	(0.053)	(0.053)
English medium school	1.020	1.029	0.340***	0.340***	-0.228***	-0.216***
	(0.132)	(0.133)	(0.047)	(0.047)	(0.053)	(0.053)
Worried about not having	0.911	0.905	0.726*	0.725*	-0.055	-0.058
sufficient food during the past month	(0.134)	(0.133)	(0.103)	(0.103)	(0.059)	(0.059)
Any chronic disease, or congenital	1.128	1.149	1.163	1.190	0.212+	0.225*
or perinatal disorder	(0.311)	(0.313)	(0.424)	(0.439)	(0.110)	(0.113)
Constant					3.138***	3.067***
					(0.091)	(0.091)
Observations	1,194	1,194	1,194	1,194	1,194	1,194
R squared					0.706	0.703

Robust standard errors in parentheses.

*** p<0.001

** p<0.01

* p<0.05

+ p<0.1

We found that higher height-for-age z-scores were associated with higher math scores, higher reading scores, and higher current grade level (**Table 2**). Likewise, stunting was associated with lower math and reading scores and lower grade level. The grade level of a stunted child was approximately one-third of a grade lower than that of a child the same age who was not stunted. In sum, consistent with the existing empirical evidence from India, we found that chronic malnutrition, as measured by stunting, was associated with a substantial reduction in cognitive achievement and educational attainment.

The association between low BMI-for-age, which is a measure of acute malnutrition, and the outcomes was less robust (**Table 3**). Higher BMI-for-age was associated with higher current grade level, and it displayed a weak positive association (statistically significant at the 10% level) with math scores. There was no association between BMI-for-age and reading scores. Thinness was associated with lower math scores at the 10% level, but not with reading scores or the grade level. In sum, the strongest evidence we uncovered regarding acute malnutrition was for the relationship between BMI-for-age and grade level, and BMI-for-age and thinness were weakly associated with math scores. There was no relationship with either measure of acute malnutrition and reading scores.

The third indicator of child nutritional status that we considered was weight-for-age, which can reflect short-term fluctuations in behavior (such as diet) and health conditions (such as diarrhea) as well as longer-term malnutrition. Higher weight-for-age was associated with higher math and reading scores and grade level (**Table 4**; the result for reading scores was significant at the 10% significance level, however). Underweight status was associated with lower reading and math scores and lower grade level. The grade level of an underweight child was approximately one-fourth of a grade lower than that of a child who was not underweight.

The results above remained unchanged with respect to coefficient sizes and significance levels when we clustered the standard errors at the level of the village instead of at the household level. This could be because children in our sample are scattered across 489 villages, with an average of only 2.4 households per village.

In terms of other determinants of cognitive achievement, child age was associated with higher reading and math scores and grade level, as expected. According to the all-India ASER assessment of rural children in 2018, girls aged 8–11 outperformed boys in reading; in math, boys scored higher than girls, with the exception of some states, including Tamil Nadu [26]. The gender gap in performance favoring girls is also apparent in our sample, as girls had significantly higher reading and math scores than boys, although in some models, girls’ current grade level was lower than boys’. Interestingly, the ASER assessment also found that the gender gap in Tamil Nadu lessened among adolescents aged 14–16, with girls maintaining a slight advantage [26]. We also tested for gender differences in the effects of nutritional status; however, we found no significant differences between boys and girls in the association between the six nutritional status indicators and the three outcomes (results not shown).

Father’s education did not appear to influence cognitive achievement, and income was positively associated with reading scores only. Mother’s education had a visible influence on math and reading scores. In particular, children of mothers with post-secondary education had significantly higher math and reading scores compared to children of mothers with lower levels of education. Mother’s education had a weak negative association (significant at the 10% level) with grade level in some models.

## Discussion

In rural Tamil Nadu, India, the poor nutritional status of children aged 8–11 appears to be strongly associated with children’s math and reading scores and current grade level. Specifically, we found that lower height-for-age and weight-for-age were associated with lower math scores, lower reading scores, and lower grade level. Lower BMI-for-age was associated with lower grade level and marginally associated with lower math scores. When we examined the binary measures of nutritional status, stunting and underweight were negatively associated with all three outcomes we examined. Thinness was associated primarily with lower math scores. The findings imply that reducing both chronic and acute malnutrition can help improve children’s cognitive achievement significantly and potentially improve grade progression.

We must interpret our findings with a number of caveats. Although we controlled for a range of potential confounders in our analysis, the observed associations in our cross-sectional study cannot be interpreted as causal. The odds ratios and coefficients in the bivariate and multivariable regressions were similar, suggesting that bias from unobserved confounding was likely low. Nonetheless, there could be omitted variables—such as home environment and developmental age, for example—for which we could not control. Furthermore, as with any study, one needs to be cautious when extrapolating the findings to other settings. While the socioeconomic and demographic characteristics of the households in our study area are representative of Tamil Nadu and South India more generally, the relationships we uncovered could be different from other settings within India and globally.

Despite these limitations, our study provides additional evidence of the centrality of malnutrition to child’s cognitive achievement and educational attainment. In particular, our study findings underscore the importance of the nutrition-cognition nexus in middle childhood. Our results support earlier findings in India [22,23] and elsewhere [18,19] on the relationship between chronic malnutrition and cognitive achievement in children above age five. Thus, lower performance in middle childhood could reflect children’s early-life conditions, including malnutrition.

We also found that acute nutritional status was associated with lower math scores and lower educational attainment, suggesting that *current* adverse conditions are also important determinants of cognitive achievement and educational attainment. There are far fewer studies of acute malnutrition than chronic malnutrition and its effects on child cognitive development [42], and those completed tend to focus on children under five and severe acute malnutrition [43]. Thus, our study contributes new findings among school-aged children. Why acute malnutrition is related to math scores and grade level and not to reading scores is an area for further research.

Our study identifies a number of additional areas for future research. First, our ability to thoroughly examine the heterogeneous effects of nutritional status on cognitive achievement and educational attainment as children age was limited by the relatively small sample size. The examination of such effects would be a natural next step in this line of research with important policy implications given India’s history of discrimination and unequal access to resources based on gender, caste, and economic status. Second, the mechanisms through which low weight-for-age and thinness—representing acute or cumulative malnutrition—influence cognitive achievement also warrants further research.

## Conclusion

Many low- and middle-income countries continue to grapple with stubbornly high rates of childhood malnutrition, which can have long-lasting effects on wellbeing. Our study concludes that a major mechanism by which nutritional deficiencies in childhood impact later life is likely through poor cognition and low educational attainment. The negative associations between measures of malnutrition and cognitive ability and educational attainment that we uncovered suggest that policies need to address both early and middle childhood and both acute and chronic malnutrition. Our findings speak to the merits of malnutrition mitigation programs, such as school lunch schemes and improved sanitation and health services.

## Ethics approval and consent to participate

This study was approved by the Institutional Review Boards of the Pennsylvania State University, USA, and the Christian Medical College, Tamil Nadu, India.

## Supporting information

S1 Table Bivariate regression results. Panel A. Bivariate regression models of the association between cognitive achievement and educational attainment and height-for-age z-scores and stunting. Panel B. Bivariate regression models of the association between cognitive achievement and educational attainment and BMI-for-age z-scores and thinness. Panel C. Bivariate regression models of the association between cognitive achievement and educational attainment and weight-for-age z-scores and underweight.
(DOCX)
