# Supplementary material for: Nutritional status, cognitive achievement, and educational attainment of children aged 8-11 in rural South India
Source: PLoS One. 2019 Oct 9;14(10):e0223001. doi: 10.1371/journal.pone.0223001 (PMC6784908; doi:10.1371/journal.pone.0223001)
Supplement: S1 Table — Panel A. Bivariate regression models of the association between cognitive achievement and educational attainment and height-for-age z-scores and stunting. Panel B. Bivariate regression models of the association between cognitive achievement and educational attainment and BMI-for-age z-scores and thinness. Panel C. Bivariate regression models of the association between cognitive achievement and educational attainment and weight-for-age z-scores and underweight. (DOCX) [file pone.0223001.s001.docx]

**Supporting information**

**Panel A. Bivariate regression models of the association between cognitive achievement and educational attainment and height-for-age z-scores and stunting**

| Outcome 🡪 | Math score | | | Reading score | | | | Grade level | | | | | | | |
| --- | --- | --- | --- | --- | --- | --- | --- | --- | --- | --- | --- | --- | --- | --- | --- |
|  | Odds ratios | | | Odds ratios | | | | OLS regression coefficients | | | | | | | |
| Height-for-age | 1.130* |  | 1.088+ | |  | | 0.050 | | |  | | | | |  |
|  | (0.056) |  | (0.054) | |  | | (0.036) | | |  | | |  |  |  |
| Stunted |  | 0.676+ |  | | 0.796 | |  | | 0.040 | | |  |  |  |  |
|  |  | (0.137) |  | | (0.142) | |  | | (0.139) | | |  |  |  |  |
| Constant |  |  |  | |  | | 4.489*** | | 4.456*** | | |  |  |  |  |
|  |  |  |  | |  | | (0.042) | | (0.038) | | |  |  |  |  |
| Observations | 1,194 | 1,194 | 1,194 | | 1,194 | | 1,194 | | 1,194 | | | | |  |  |
| R squared |  |  | |  | |  | | 0.002 | | | 0.000 | | | | |

**Panel B. Bivariate regression models of the association between cognitive achievement and educational attainment and BMI-for-age z-scores and thinness**

| Outcome 🡪 | | Math score | | | | Reading score | | | | | | | Grade level | | | | | | | | | |
| --- | --- | --- | --- | --- | --- | --- | --- | --- | --- | --- | --- | --- | --- | --- | --- | --- | --- | --- | --- | --- | --- | --- |
|  | Odds ratios | | | | | | Odds ratios | | | | | OLS regression coefficients | | | | | | | | | |  |
| BMI-for-age | | 1.110** |  | | 1.047 | | | |  | | | 0.029 | | |  | | | | |  |  |  |
|  | | (0.043) |  | | (0.041) | | |  | | | (0.027) | | |  | | | |  |  |  |  |  |
| Thinness | |  | 0.775+ | | |  | | | | 0.852 | | |  | | | | 0.099 | |  |  |  |  |
|  | |  | (0.103) | | |  | | | | (0.107) | | |  | | | | (0.087) | |  |  |  |  |
| Constant | |  |  | | |  | | | |  | | | 4.489*** | | | 4.436*** | | | | |  |  |
|  | |  |  | | |  | | | |  | | | (0.047) | | | | (0.043) | | | | | |
| Observations | | 1,194 | 1,194 | | 1,194 | | | | 1,194 | | | 1,194 | | | 1,194 | | | | |  |  |  |
| R squared | |  | |  | |  | | | |  | | | 0.001 | | | | 0.001 | | | | | |

**Panel C. Bivariate regression models of the association between cognitive achievement and educational attainment and weight-for-age z-scores and underweight**

| Outcome 🡪 | Math score | | | Reading score | | | | | | Grade level | | | | | | | | |  |  |
| --- | --- | --- | --- | --- | --- | --- | --- | --- | --- | --- | --- | --- | --- | --- | --- | --- | --- | --- | --- | --- |
|  | Odds ratios | | | Odds ratios | | | | | | OLS regression coefficients | | | | | | | | |  |  |
| Weight-for-age | 1.187*** | |  | | 1.109* | | |  | | | | 0.112*** | | | | |  | | | |
|  | (0.051) | |  | | (0.048) | |  | | | | (0.029) | | | | |  | | | |  |
| Underweight |  | 0.598*** | |  | | 0.676** | | |  | | | | -0.224* | | | | |  |  |  |
|  |  | (0.077) | |  | | (0.083) | | |  | | | | (0.090) | | | | |  |  |  |
| Constant |  |  | |  | |  | | | | 4.582*** | | | | 4.516*** | | | |  |  |  |
|  |  |  | |  | |  | | | | (0.050) | | | | (0.043) | | | |  |  |  |
| Observations | 1,194 | 1,194 | | 1,194 | | 1,194 | | | | 1,194 | | | | | 1,194 | | | |  |  |
| R squared |  |  | |  | |  | | | | 0.012 | | | | | 0.005 | | | |  |  |

Notes: In all panels, robust standard errors in parentheses. *** p<0.001, ** p<0.01, * p<0.05, + p<0.1
